# Choice-Induced Preference Change in the Free-Choice Paradigm: A Critical Methodological Review

**DOI:** 10.3389/fpsyg.2013.00041

**Published:** 2013-02-07

**Authors:** Keise Izuma, Kou Murayama

**Affiliations:** ^1^Division of Humanities and Social Sciences, California Institute of TechnologyPasadena, CA, USA; ^2^Brain Science Institute, Tamagawa UniversityTokyo, Japan; ^3^Department of Psychology, University of CaliforniaLos Angeles, CA, USA

**Keywords:** preference change, attitude change, free-choice paradigm, cognitive dissonance, choice justification, self-perception theory, cognitive consistency, social influence

## Abstract

Choices not only reflect our preference, but they also affect our behavior. The phenomenon of choice-induced preference change has been of interest to cognitive dissonance researchers in social psychology, and more recently, it has attracted the attention of researchers in economics and neuroscience. Preference modulation after the mere act of making a choice has been repeatedly demonstrated over the last 50 years by an experimental paradigm called the “free-choice paradigm.” However, Chen and Risen (2010) pointed out a serious methodological flaw in this paradigm, arguing that evidence for choice-induced preference change is still insufficient. Despite the flaw, studies using the traditional free-choice paradigm continue to be published without addressing the criticism. Here, aiming to draw more attention to this issue, we briefly explain the methodological problem, and then describe simple simulation studies that illustrate how the free-choice paradigm produces a systematic pattern of preference change consistent with cognitive dissonance, even without any change in true preference. Our stimulation also shows how a different level of noise in each phase of the free-choice paradigm independently contributes to the magnitude of artificial preference change. Furthermore, we review ways of addressing the critique and provide a meta-analysis to show the effect size of choice-induced preference change after addressing the critique. Finally, we review and discuss, based on the results of the stimulation studies, how the criticism affects our interpretation of past findings generated from the free-choice paradigm. We conclude that the use of the conventional free-choice paradigm should be avoided in future research and the validity of past findings from studies using this paradigm should be empirically re-established.

## Introduction

Individuals not only behave according to their preference (e.g., “I choose it because I like it”), but their choice behavior also affects their preference (e.g., I like it because I chose it). This process of choice-induced preference change is traditionally explained by cognitive dissonance theory (Festinger, 1957; or by self-perception theory, see Bem, 1967). When there is inconsistency between preference and behavior (i.e., choosing something I don’t like), it causes an uncomfortable feeling called “cognitive dissonance,” which in turn motivates a person to modulate his preference in order to restore the consistency. Over the last six decades, preference/attitude change following the mere act of making a choice has been repeatedly demonstrated through the free-choice paradigm (Brehm, 1956). In this paradigm, participants are first asked to rate (or rank) several items (e.g., music albums, posters, foods, political candidates, jobs, etc.) according to their preference (first rating task). Second, they are asked to choose between two of the items that had similar preference ratings in the first rating task (choice task). Finally, they are asked to rate their preference for the same items one more time (second rating task). Studies found that after making a difficult choice between two equally preferred items, participants’ preference for the chosen item increases while preference for the rejected item decreases (i.e., so-called “spreading of alternative”; e.g., Brehm, 1956; Gerard and White, 1983; Steele et al., 1993; Heine and Lehman, 1997).

Because the phenomenon of choice-induced preference modulation challenges a vital assumption in neoclassical economics that people’s behavior is determined by preference, it has attracted the attention of researchers from various disciplines including psychology, economics, and neuroscience (Ariely and Norton, 2008; Leotti et al., 2010). Studies using the free-choice paradigm, have involved healthy adult humans as well as amnesia patients (Lieberman et al., 2001), 4-year-old children and capuchin monkeys (Egan et al., 2007, 2010). More recently, the brain mechanisms underlying choice-induced preference modulation have been extensively studied with the same paradigm (Sharot et al., 2009, 2010a; Izuma et al., 2010; Jarcho et al., 2011; Qin et al., 2011; Kitayama et al., 2013).

In 2010, however, more than a half century after the original study by Brehm (1956), Chen and Risen pointed out an important methodological problem in the free-choice paradigm, and they argued that all findings from the studies using the free-choice paradigm are inconclusive (Chen and Risen, 2010; see also Risen and Chen, 2010). The original paper describing the methodological flaw was made available to the public as a working paper in 2008 and attracted the attention of researchers (see Chen and Risen, 2009; Sagarin and Skowronski, 2009a,b). However, despite the fact that their critique could potentially undermine the conclusions of any study that uses the paradigm, behavioral, and neuroimaging studies using the paradigm continue to be published without addressing the critique (Sharot et al., 2009, 2010a; Coppin et al., 2010, 2012; Imada and Kitayama, 2010; Lee and Schwarz, 2010; West et al., 2010; Harmon-Jones et al., 2011; Jarcho et al., 2011; Qin et al., 2011; Kimel et al., 2012; Kitayama et al., 2013). Furthermore, although some researchers have already provided evidence for the existence of choice-included preference change using new paradigms or modifications of the free-choice paradigm, some of them are not sufficiently compelling, as detailed later.

In this article, we first briefly describe the methodological flaw raised by Chen and Risen (2010). Second, in order to help readers intuitively understand the problem, we report stimulation studies that highlight how the free-choice paradigm measures ostensible preference change without any change in true preference. Furthermore, our stimulation study also shows how noise levels in each phase of the free-choice paradigm independently affect the artificial spreading of alternatives. These simulation studies provide important background knowledge to help evaluate why some methods are better than others in addressing the critique and why some past findings are more vulnerable to the critique than others. Third, based on the results of the stimulation studies, we review and discuss ways of addressing the problem as done in some recent studies. We also conducted a meta-analysis to examine how the criticism affects the effect size of choice-induced preference change. Finally, we review how detrimental this criticism could be to specific findings from past studies that used the free-choice paradigm.

## The Methodological Flaw in the Free-Choice Paradigm

The main claim of Chen and Risen is that the original free-choice paradigm could produce predicted preference change (“spreading of alternatives”) even without any change in true preference (that is, without any experience of cognitive dissonance) because the participants’ choice during the choice task has additional information about their preference, which can bias measured preference change (Chen and Risen, 2010).

**Figure 1:**
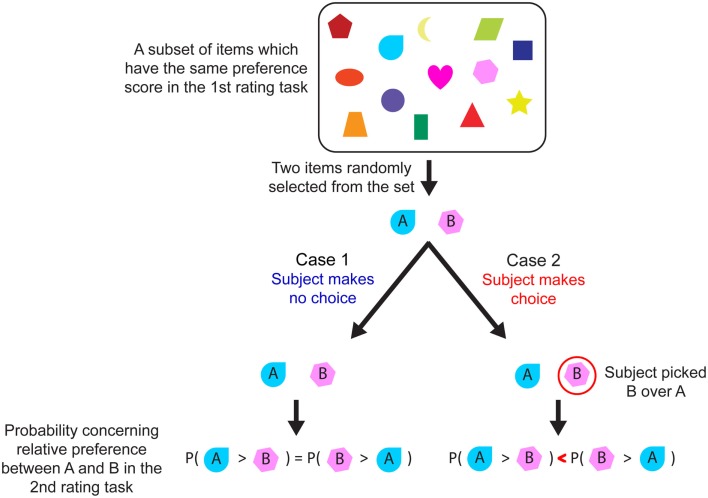
**Schematic illustration of the methodological flaw in the free-choice paradigm**. Each object in the box represents an item (e.g., music CD). If two items (A and B) were randomly selected from the set, and a participant made no choice (Case 1), the only information available about participant’s preference for items A and B is that both had the same ratings in the first rating task, and thus we cannot predict which item would be rated higher during the second rating task. In contrast (Case 2), the participant’s choice provides additional information about his/her relative preference between the two items. Thus, if a participant selected B over A, item B (chosen item) is likely to be rated higher than item A (rejected item) during the second rating task (i.e., a spread of alternatives is more likely to be observed).

Suppose a person rates 50 items according to his/her preference (first rating task), and then rates the same items again an hour later (second rating task). If we randomly pick two items (item A and item B in Figure 1) from a subset of items that had the same preference rating in the first rating task, the probability that the person’s preference for A is higher than B in the second rating task is the same as the probability that B is higher than A (see Figure 1 case 1). Because the preference rating, just like any other subjective rating, is susceptible to noise, the preference for each item varies randomly between the two rating sessions. Therefore, we cannot predict whether one’s preference will go up or down for any given item.

However, what if we know that a person had a choice between A and B, and he/she picked B over A? We would then be able to infer that the person’s true preference is at least slightly higher for B than for A, even though the ratings were equal during the first rating task. Then, knowing that this person slightly prefers B over A, it seems more likely that in the second rating task, his/her preference rating for B will be higher than that for A (Figure 1 case 2). Thus, if we had additional information about one’s true preference, as revealed by his/her direct choice between A and B, we could predict whether his/her preference rating is more likely to increase or decrease for each item in a second rating task.

This is the basis of the original free-choice experiment. Two items that had similar preference ratings in the first rating session are categorized as “chosen” or “rejected” items based on the participant’s choice between them. Then, in the second rating task, preference for a chosen item is more likely to increase than decrease, and preference for a rejected item is more likely to decrease than increase. In other words, in the free-choice paradigm, researchers do not randomly categorize items into two categories and compare them to test the effect of choice on preference. Instead, based on the information provided by participants’ choices, they systematically categorize items into those whose rating is likely to increase (chosen items) and those whose rating is likely to decrease (rejected items; i.e., choice and true preference is confounded). Thus, the free-choice paradigm could be measuring systematic preference change (i.e., spreading of alternatives) even in the complete absence of a change in true preference.

According to Chen and Risen (2010), the argument above is based on three assumptions, all of which seem to be uncontroversial: (1) ratings provide a statistically unbiased measure of a participant’s feelings about that item, (2) participants’ choices are at least partially guided by their preferences, and (3) participants’ ratings are not a perfect measure of their preferences. Chen and Risen (2010) also provided more formal mathematical proof of how the free-choice paradigm could produce systematic preference change in the absence of cognitive dissonance if the three assumptions were met (see Chen and Risen, 2010 for more details).

It should be noted that in the free-choice paradigm, preference change after a difficult choice between two items with similar preference was typically compared with preference change after an easy choice between one liked and one disliked item. Studies typically show a spreading of alternatives that is larger after a difficult choice compared to an easy choice, which is consistent with the prediction by cognitive dissonance theory (during easy choices, participants usually choose a liked item and reject a disliked item, both of which are cognitively consistent, and thus less cognitive dissonance and less preference change are expected). However, this is also what is expected by the methodological artifact because easy choices provide much less “additional” information about a participant’s true preference compared to difficult choices (see Chen and Risen, 2010). Based on the first rating, we are fairly certain which item in an easy choice pair is truly preferred, and therefore the spread of alternatives is not as heavily influenced by the information revealed by choice in the easy choice condition.

## A Simulation Demonstrating How Preference Change is Produced Without Any Change in True Preference

To illustrate the problem more concretely, we conducted a computer simulation to mimic a typical free-choice study (for details, see Appendix). One advantage of conducting a simulation is that we can monitor the true preference score (which cannot be observed in a real study) for each item throughout the hypothetical experiment. This helps us understand why the paradigm produces a positive spreading of alternatives without a change in true preference. Let us explain how the artifact is produced in a typical free-choice paradigm by following the simulation step by step.

**Figure 2:**
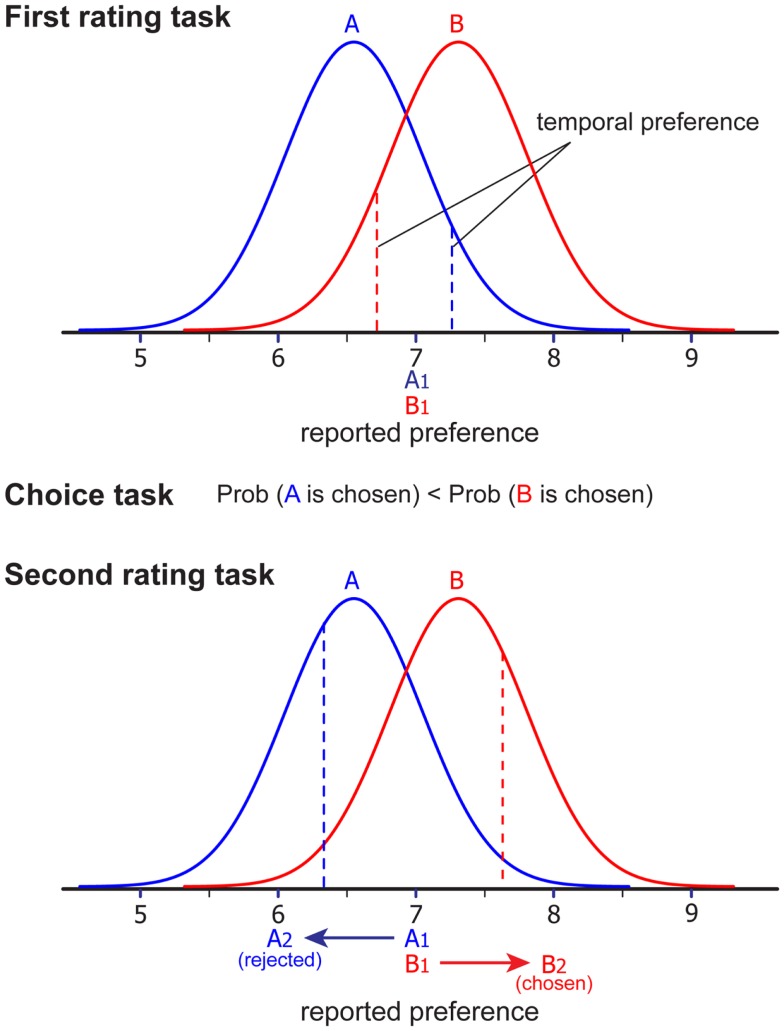
**Schematic illustration of how the free-choice paradigm produces artificial spreading of alternatives in the simulation study**. Each normal curve represents the distribution of participant’s preference (true preference + random noise) for item A (blue) and B (red). In our simulation study, participants’ temporal preference for each item was randomly drawn from each distribution, and these numbers are rounded to the nearest integer that represents participants’ reported preference for each item. In this example, reported preferences for both items are 7 (A1 = B1 = 7). When a participant is asked to make a choice between A and B in the choice task, B is more likely to be chosen because true preference for B is higher than that for A. Then, when participants’ temporal preference for each item was randomly drawn for the second time from the same distribution (preference rating task 2), temporal preference for A2 (rejected item) is likely to decrease, while temporal preference for B2 (chosen item) is likely to increase purely by chance. Accordingly, reported preference for A is also likely to increase, while reported preference for B is likely to decrease (A2 = 6, B2 = 8 in this example). Note that distributions for each item A and B stay the same across two preference rating tasks, indicating that there is no change in participants’ true preference.

In the simulation, hypothetical participants rated a set of items (first rating), then chose items among pairs (choice), and finally rated the items again (second rating). Each item was assigned a “true preference score.” Importantly, we assumed that true preference remains stable across the three phases, and therefore the spreading of alternatives found in our simulation study can never be attributed to the effect of choice or cognitive dissonance on true preference. In the first rating phase, we added random noise to the true preference score to generate a “temporal preference” score and paired the items that are closely matched. That is, we paired the items that receive the same ratings on a 10-point scale (1 = don’t like it at all; 10 = like it very much; see Figure 2, top). The important observation from Figure 2 is that, although each item in a pair received identical attractiveness ratings (reported preference), their true preference is different (i.e., the true preference for item B is higher than the true preference for item A). Because of the random noise, some item pairs could show substantial within-pair differences in the true preference scores. This difference is the key factor that produces the spurious dissonance effect identified by Chen and Risen (2010).

In the choice task, our simulation assumed that participants are more likely to choose the item with a higher true preference score (the second assumption by Chen and Risen, 2010). That is, in the current example, item B is more likely to be chosen than item A (see Figure 2, middle). With this assumption, although choice involves some noise and therefore choice is probabilistic (i.e., there are some cases where items with lower true preference are chosen), on average, chosen items have higher true preference scores than rejected items. In other words, choice based on individual preference inevitably provides some information about the underlying true preference (i.e., information revealed by choice; Chen and Risen, 2010).

In the second rating task, we again added random noise to the true preference score to generate a temporal preference score for both chosen and rejected items (Figure 2, bottom). At this point, because chosen items have higher true preference than rejected items, the chosen items are more likely to receive higher attractiveness ratings than the rejected items (Figure 2, bottom). That is, even if there is no choice-induced preference change, the typical free-choice paradigm produces an artificial spread of alternatives in the second rating task.

**Figure 3:**
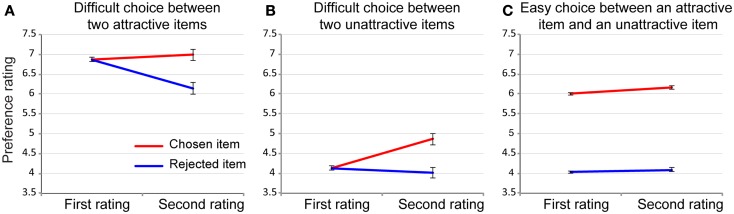
**Simulated patterns of preference change across different choice conditions (replication = 10,000)**. In this simulation, noise in both the rating and choice phase was set at 0.5 (relative magnitude in comparison to the standard deviation of the true preference; see also Figure 4). **(A)** Preference change after difficult choices between two attractive items of the same self-report preference. **(B)** Preference change after difficult choice between two unattractive items of the same self-report preference. **(C)** Preference change after easy choice between one attractive item and one unattractive item (self-report preferences differs more than two points in the 10-point scale). Error bars depict the SEM.

This hypothetical experiment (80 items, *N* = 10) was repeated 10,000 times, and the averaged results are plotted in Figure 3A. The results clearly showed that chosen items are rated as more attractive than rejected items in the second rating task. We also simulated a difficult choice between two equally unattractive items (Figure 3B) and an easy choice between one attractive item and one unattractive item (Figure 3C). Consistent with Chen and Risen (2010), easy choice pairs did not produce a noticeable spread of alternatives (Figure 3C). Unattractive pairs produced a similar spread of alternatives as did attractive pairs (Figure 3B). Thus, the more difficult the choice, the larger the spreading of alternatives produced by the artifact. An interesting observation is that, when choices are made between two attractive items, the spread of alternatives is largely driven by the decreased preference for the rejected items (Figure 3A), whereas when choices are made between two unattractive items, the spread of alternatives is largely driven by the increased preference for the chosen items (Figure 3B).

This pattern of preference change is what the cognitive dissonance theory predicts because choosing an attractive item and rejecting an unattractive item are both more cognitively consistent and thus induce much less cognitive dissonance (and less preference change) than choosing an unattractive item or rejecting an attractive item. In other words, our simulation showed that the pattern of ostensible preference change produced by the artifact exactly matches the pattern predicted by cognitive dissonance theory (Festinger, 1957), highlighting how deep-rooted this problem is in cognitive dissonance research with the free-choice paradigm. In fact, this pattern is also largely consistent with most of past empirical findings (e.g., Brehm, 1956; Vroom, 1966; Gerard and White, 1983; Shultz et al., 1999; Harmon-Jones and Harmon-Jones, 2002).

Note that there are two major parameters that influence the spurious dissonance effect. The first parameter is the magnitude of the random noise in the two rating phases. This parameter has two opposing effects. Random noise in the first rating task expands the difference in true preference within matched pairs presented in the choice phase. Accordingly, larger random noise increases the difference between the chosen and rejected items (i.e., the larger the random noise in the first rating, the larger the spreading of alternatives). On the other hand, random noise in the second rating task works to mask the difference in the true preference inherent in chosen and rejected items (i.e., the larger the random noise in the second rating, the smaller the spreading of alternatives). As a result, the magnitude of the random noise in rating is expected to have an inverted-U shaped effect on the spreading of alternatives (i.e., extremely small or large random noise reduces the spreading of alternatives by the artifact). The second parameter is the magnitude of the random noise in the choice phase. This parameter basically reduces the difference between chosen and rejected items, as true preference would have less influence on choice with larger random noise (Chen and Risen, 2009; Sagarin and Skowronski, 2009a).

**Figure 4:**
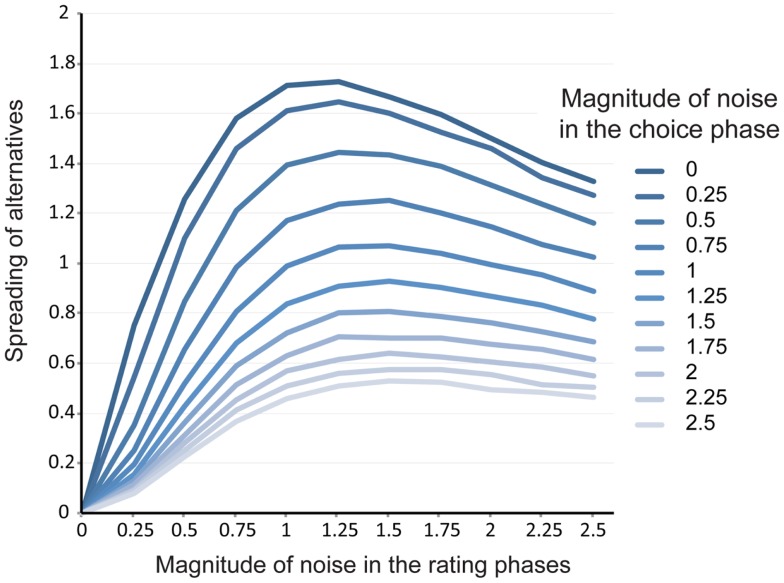
**Simulated magnitude of spreading of alternatives by the artifact as a function of noise in the two rating and choice phases (replication = 10,000)**. Spreading of alternatives is computed as (second rating for a chosen item – first rating for a chosen item) – (second rating for a rejected item – first rating for a rejected item). Noise is represented as the relative magnitude in comparison to the standard deviation of the true preference (i.e., SD = 2).

To quantify the effects of these parameters, we conducted another simulation that independently manipulated the magnitude of each random noise. Figure 4 shows the spreading of alternatives as a function of each random noise. As expected, random noise in the rating phase had a curve linear effect, whereas random noise in choice had a negative monotonic effect on the spreading of alternatives. This result specifies a boundary condition on the extent to which the criticism made by Chen and Risen (2010) is consequential. It should be noted that our simulation employed a subjective rating as a measure of preference, but it can be easily generalized to other experimental situations. For example, several previous studies used a ranking paradigm in which participants rank items according to their preference (e.g., Gerard and White, 1983; Lieberman et al., 2001; Kitayama et al., 2004; Lee and Schwarz, 2010). Assuming that ranking is (at least partly) guided by true preference, such a ranking paradigm would also produce the same pattern of results. For the same reason, other measures of preference are also susceptible to the artifact (affective priming task: Gawronski et al., 2007; brain activations: Sharot et al., 2009; Sharot et al., 2010a). In addition, the basic findings would not be influenced by other factors such as coarseness of rating scale (e.g., whether a 5-point or a 100-point scale was used; see Appendix) or number of items presented, as these factors are essentially irrelevant to the information revealed by choice.

## How Can the Problem be Addressed?

After Chen and Risen’s (2010) critique spread among the scientific community, researchers began to seek new methodologies to address the problem. So far, three main methods have been proposed^1^ (see also Risen and Chen, 2010). (1) Set up a choice task so that participants’ choices do not reflect their preference (blind choice paradigm; Egan et al., 2010; Sharot et al., 2010b), (2) measure the degree of preference change caused purely by the artifact and subtract it from preference change observed in the typical free-choice paradigm (rate-rate-choose paradigm; Chen and Risen, 2010; Izuma et al., 2010; Sharot et al., 2012), and (3) measure preference changes for two items that are not compared in a direct choice between them, but rather through other comparisons (implicit choice paradigm; Alós-Ferrer et al., 2012). Here, we review studies using each of these methods and how successful each method is in addressing the problem. We also run a meta-analysis to see the effect size of choice-induced preference change after the criticism is properly addressed.

### Blind choice paradigm

Egan et al.’s (2010) tried to demonstrate whether 4-year-old children and capuchin monkeys show choice-induced preference change by fixing the methodological flaw in their original study (Egan et al., 2007). In their new paradigm (Egan et al., 2010), they had children blindly make a choice (Experiment 1; i.e., making choice without knowing objects’ identities. This method was first suggested by Sagarin and Skowronski (2009a)) or gave monkeys an illusion of choice (Experiment 2; although monkeys felt that they themselves selected an object, in reality the object had been randomly assigned by the experimenter). The point here is that in both cases, the choice did not reflect the children’s or monkeys’ preference so that Chen and Risen’s (2010) second assumption no longer holds. As suggested in our simulation study (see Figure 4), if choice did not reflect participant’s preference (e.g., large noise in the choice phase), the paradigm does not produce artificial spreading of alternatives so that the pure effect of choice (dissonance) on preference can be tested (Sagarin and Skowronski, 2009a). It should be noted, however, that although these are elegant ways to address the critique, their study itself (i.e., Egan et al., 2010) has other shortcomings, which make it difficult to interpret their findings (see Risen and Chen, 2010). Specifically, in Egan et al. (2010) first experiment, in order to measure how children’s preference changed after the blind choice, they had children make another blind choice. Unfortunately, this cannot be a good measure of preference because as stated above, blind choice does not reflect preference. Furthermore, in their second experiment with capuchin monkeys, monkeys’ preference after illusory choice was measured by having them make 10 (non-blind) choices. However, if choice could modulate preference, it is very likely that preference can be modulated dynamically during these 10 choices, and preference for each object can never be correctly measured (see Risen and Chen, 2010 for more details, see also Holden, 2013). Therefore, although each of their new manipulations of choice (blind choice and an illusion of choice) adequately addresses the problem, the findings are not sufficiently compelling.

A similar blind choice method was used by Sharot et al. (2010b). In this study, during the choice task, participants were presented with two items (vacation destinations) only very briefly (2 ms). Furthermore, the stimuli presented during this 2 ms were actually nonsense strings of letters. Therefore, participants’ choices during this blind choice task never reflected their preference, and choices could never provide any information about participants’ preference. Interestingly, the study found that participants’ preference for stimuli they thought they had selected during the blind choice task significantly increased while no preference change was observed in the control condition where a computer randomly made the choices (Sharot et al., 2010b).

### Rate-rate-choose paradigm

The second strategy was proposed in Chen and Risen’s (2010) original paper, and they included a new control condition that is influenced by the artifact but not by the choice-induced preference change. The idea here is that changes in true preference can be tested by comparing preference changes in the typical free-choice paradigm with preference changes produced only by the artifact. Whereas in the typical paradigm, participants performed the first rating task, the choice task, and then the second rating task (Rate-Choose-Rate order), in their new control condition, participants performed the choice task after two rating tasks (i.e., Rate-Rate-Choose order)^2^. In this Rate-Rate-Choose condition, any preference change between the two rating tasks can never be attributed to the effect of making a choice (or cognitive dissonance). On the other hand, the choice task still can provide information about the participants’ true preference even if the task is performed at the end, and thus it could predict preference change. Supporting their argument, they first found that the Rate-Rate-Choose condition could measure predicted spreading of alternatives (Chen and Risen, 2010), thus experimentally demonstrating that the free-choice paradigm could measure spreading of alternatives even if the participants’ true preferences remain completely stable. Because preference change measured in the Rate-Rate-Choose condition reflects preference change explained only by the artifact, the existence of choice-induced preference change could be tested by comparing the Rate-Choose-Rate vs. Rate-Rate-Choose conditions. Their first study failed to find any evidence of choice-induced preference change above and beyond the artifact. However, after modifying the experimental procedure so that more dissonance was thought to be induced (i.e., making choice more important and meaningful), they found a marginally significant difference between preference change in the Rate-Choose-Rate condition and that in the Rate-Rate-Choose condition (*p* = 0.06; Chen and Risen, 2010), suggesting that the mere act of making a choice seems to have an effect on preference over and above the artifact.

Similarly, Izuma et al. (2010) conducted a functional magnetic resonance imaging (fMRI) study in which the criticism was addressed by employing the Rate-Rate-Choose condition as suggested by Chen and Risen (2010). The study found that preference change in the Rate-Choose-Rate condition (self-difficult condition in the original paper) was significantly larger than that in the Rate-Rate-Choose condition (post-ex choice condition; Izuma et al., 2010). Furthermore, basically the same pattern of preference change was observed in the activity of the reward-related brain area (i.e., ventral striatum; Izuma et al., 2010), thus providing equivocal evidence for the existence of choice-induced preference change. Making choices affects not only self-report preference but also its neural representation.

More recently, using the Rate-Rate-Choose condition as a control condition, Sharot et al. (2012) measured preference change immediately after participants made choices, as well as 3 years later, to test the duration of choice-induced preference change. In order to control the effect of the artifact on long-lasting preference change, they asked participants to perform the choice task on the first day (Rate-Choose-Rate condition) as well as 3 years later, after the second rating task (Rate-Rate-Choose condition). Although the study addresses the criticism to some extent, we have two concerns. First, the timing of the choice tasks of two conditions was quite different (the choice in the Rate-Rate-Choose condition was made 3 years after the first session). Thus, it is unclear whether the level of noise is the same between two choice tasks, which makes it difficult to interpret the difference between the Rate-Choose-Rate and Rate-Rate-Choose conditions (see Figure 4 for how a different level of noise in the choice task affects the magnitude of preference change by the artifact). Because it is unclear how a 3-year interval affects the level of noise in the rating and choice phases, the Rate-Rate-Choose condition would not be a good control condition, especially for testing the long-term effect of choice on preference. Second, while there was a significant difference between the Rate-Choose-Rate and the Rate-Rate-Choose conditions in the first session (immediate preference change), a significant difference between these two conditions was not reported in the second session (long-lasting preference change).

### Implicit choice paradigm

The third strategy (Alós-Ferrer et al., 2012) aims to demonstrate choice-induced preference change using an implicit choice method. In this study, participants first rated 80 items, made choices between item pairs, and then rated all of them again. However, unlike the typical free-choice paradigm, two items with the same reported preference (*a* and *b*) were categorized as “chosen” or “rejected” on the basis of a choice between each of the two items and another item, not by direct comparison (i.e., “implicit choice” between *a* and *b*). That is, items *a* and item *b* were each paired with another item (*h* or *l*), and participants made choices between *a* and *h*, and *b* and *l*. Items *h* and *l* were selected from a set of items based on the first rating; reported preference for *h* was higher than *a*, and reported preference for *l* was lower than *b* (*h* > *a* = *b* > *l* in the first preference rating). Therefore, participants were likely to choose *h* over *a*, and *b* over *l*. Then, preference change between the first and second rating were compared between the rejected item *a* and the chosen item *b*. Importantly, if one of choices in the *a-h* and *b-l* pairings was not as expected, the four items were excluded from the analysis (i.e., selection bias).

Unfortunately, although the information about relative preference between *a* and *b* was never revealed by a direct choice, we think that this method is still susceptible to the artifact to some extent^3^. First, although participants did not make a direct choice between *a* and *b*, choices between *a* and *h*, and *b* and *l* can still reveal some information about true preference for *a* and *b*. For example, take an extreme case in which the four items (*a*, *b*, *h*, and *l*) have the same preference rating in the first rating task (*h* = *a* = *b* = *l*). If participants picked *h* over *a*, and *b* over *l*, the second preference ratings for rejected items (*a* and *l*) are likely to decrease while ratings for chosen items (*b* and *h*) are likely to increase. Thus, although no choice was made between *a* and *b*, we can still predict that the second rating for *b* is likely to be higher than *a* (i.e., spread of alternatives). Just like the differential level of artificial spreading of alternatives for easy and difficult choice conditions seen in our simulation (see Figure 3), the amount of information about a participant’s true preference that choices can reveal depends on how close the first preference ratings for each item in a pair (*a-h* or *b-l* pair) were (i.e., how difficult the choice is). The closer the reported preferences between *a* and *h*, and *b* and *l*, the more additional information choices can reveal, and thus the larger the spread of alternatives by the artifact. This seems to fit with their findings that spreading of alternatives generally decreases as the difference (D) in reported preference between *a* and *h*, and *b* and *l* increases, thus suggesting that the implicit choice method is susceptible to the artifact.

It should be noted, however, that they also conducted an additional analysis that they called a “robustness check.” In this analysis, they included all items regardless of the participant’s actual choices by treating them as if all of choices were as expected from the first rating (item *a* as rejected and item *b* as chosen). Because there is no selection bias in this case, the artifact should not produce preference. In other words, the findings from the implicit choice method are considered to be valid only for the results from this robustness check analysis. Alós-Ferrer et al. (2012) revealed significant preference change with this analysis. Thus, although implicit choice is not an ideal method to deal with the artifact, it nonetheless could provide evidence for choice-induced preference change. One weakness of the robustness check analysis is that the exact effect size of choice-induced preference change cannot be estimated, as the analysis considers some rejected items as chosen ones (or *vice versa*).

### Meta-analysis and summary

**Figure 5:**
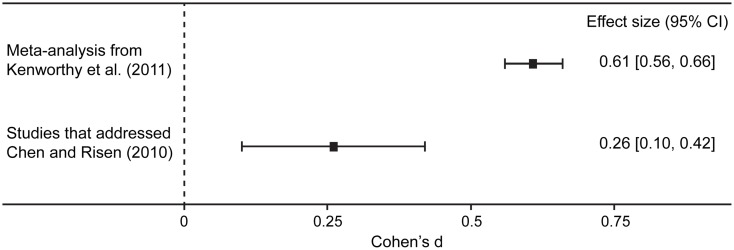
**Forest plot that compares the averaged effect size reported in a previous meta-analysis (Kenworthy et al., 2011; *k* = 18) and that reported in the studies addressing Chen and Risen’s (2010) criticism (*k* = 4)**.

It is worth noting that the magnitude of the effect is substantially smaller than that reported in previous studies when researchers appropriately used these methodologies to address Chen and Risen’s (2010) critique. In fact, in both Sharot et al. (2010b) and Izuma et al. (2010), the effects are significant only with one-tailed tests (i.e., 0.05 < two-tailed *p*-values < 0.10). The behavioral experiments conducted by Chen and Risen (2010) exhibited non-significant and marginally significant effects. To quantify the magnitude of the choice-induced preference change, we meta-analyzed the effect sizes of these studies.^4^ Izuma et al. (2010) and Sharot et al. (2010b) utilized a matched-group experimental design, comparing the spreading of alternatives of an experimental condition with that of an appropriate control condition (i.e., blind choice or Rate-Rate-Choose condition; see the previous section). In this case, we would need correlation between spreading of alternatives of the experimental condition and those of the control condition to estimate effect size (Dunlap et al., 1996). This information is not available from Sharot et al. (2010b). Accordingly, we used the correlation coefficient obtained from Izuma et al. (2010) to compute the effect size of Sharot et al. (2010b). The effect sizes for these studies did not significantly vary across the studies, *Q*(3) = 0.618, *p* = 0.89. Importantly, the integrated effect size is statistically significant, but small in magnitude based on the conventional criterion (Cohen, 1988), *d* = 0.26, 95% CI = (0.10, 0.42). This effect size is also substantially smaller than that reported in the recent meta-analysis, *d* = 0.61, 95% CI = (0.55, 0.66; Kenworthy et al., 2011; see Figure 5). These findings indicate that choice-induced preference change does exist, but past studies substantially overestimated the effect due to the methodological artifact pointed out by Chen and Risen (2010).

So far there are three ways of addressing the criticism, and each has produced evidence that choice-induced preference change truly exists. However, the effect size is considerably smaller than what had been previously shown. It is also important to note that not all studies using these methods provided sufficient evidence. As stated above, the Rate-Rate-Choose condition is probably not a good way to control the effect of information revealed by choice especially when testing for long-lasting preference change. Furthermore, although Alós-Ferrer et al. (2012) tried to address the problem, their implicit choice paradigm seems to be susceptible to the artifact as well, illustrating how complicated the problem is. There could be other good methods of tackling the problem, but researchers should carefully examine the validity of a new paradigm before conducting a study. We hope that the results of our simulation study (see Appendix for details) will help researchers judge the validity of their own method, and perhaps encourage them to run their own simulation to test the validity of a new paradigm before conducting experiments.

## How Does the Problem Affect Past Findings?

### How serious is the problem?

It is important to note that Chen and Risen’s (2010) argument says nothing about whether choice-induced preference change is real or not. Rather, their claim is that the preference change measure in the typical free-choice paradigm is confounded with an artifact for any particular study. As our brief review and meta-analysis indicate, choice-induced preference change has been observed after properly addressing the criticism by Chen and Risen (2010). These findings suggest that choice-induced preference change does exist, at least under certain conditions. However, this does not mean that the criticism was invalid or unimportant. Three studies (Chen and Risen, 2010; Izuma et al., 2010; Sharot et al., 2012) demonstrated that the free-choice paradigm could measure significant preference change without any change in true preference (i.e., significant preference change in the Rate-Rate-Choose condition), which indicates that the confounding effect is serious and non-negligible. In fact, our meta-analysis shows that the effect size for choice-induced preference change is considerably smaller than what was previously shown. In addition, our simulation indicated that the degree of confounding varies depending on several factors that are specific to individual experiments (see also Sagarin and Skowronski, 2009a). Therefore, the fact that some well-crafted studies (e.g., Chen and Risen, 2010; Izuma et al., 2010; Sharot et al., 2010b; Alós-Ferrer et al., 2012; Johansson et al., 2012) provided evidence for choice-induced preference change does not mean that other studies are immune to the confounding issue. The confounding of the artifact must be taken into account in future studies of choice-induced preference change, and the results from past studies that did not address the issue must be re-established.

Therefore, at least some of results of past studies using the traditional free-choice paradigm are in question. In addition to the original study by Brehm (1956), investigations of the detailed pattern of choice-induced preference change (Greenwald, 1969; Gerard and White, 1983; Shultz et al., 1999) and studies that simply aimed to demonstrate choice-induced preference change using different attitude objects such as jobs (Vroom, 1966; Lawler et al., 1975) or olfactory stimuli (Coppin et al., 2010, 2012) are the most vulnerable to the criticism.^5^ Similarly, as stated above, the same criticism applies to studies using a different measure of preference, such as implicit association between a target item and positive/negative words (Gawronski et al., 2007) or brain activations (Sharot et al., 2009, 2010a). In what follows, we will briefly review how the criticism affects other findings.

### Studies investigating the effect of memory about past choices

The effect of explicit memory about past choice has been investigated by comparing normal control participants with amnesic patients (Study 1 in Lieberman et al., 2001) or by measuring participants’ memory about their previous choices (Coppin et al., 2010, 2012). However, as the Rate-Rate-Choose condition could produce a significant artificial preference change (Chen and Risen, 2010; Izuma et al., 2010; Sharot et al., 2012), the original free-choice paradigm could measure ostensible preference change without explicit memory of choices simply due to the artifact (i.e., in the Rate-Rate-Choose condition, participants had absolutely no memory of their choices during the second rating task simply because they had not made any choices yet). Thus, preference changes reported in all of these studies are possible even without any change in true preference following choice.

### Studies investigating the effect of other moderating factors

Using the free-choice paradigm, many social psychological studies have investigated the effect of moderating factors on choice-induced preference change as a measure of dissonance reduction. The studies typically compare preference changes between two or more conditions with different experimental manipulations (e.g., choice importance, choice reversibility, etc.; e.g., Brehm, 1956; Brehm and Cohen, 1959; Deutsch et al., 1962; Brock, 1963; Walster et al., 1967; Greenwald, 1969; Brehm and Jones, 1970; Brehm and Wicklund, 1970; Gordon and Glass, 1970; Walster and Walster, 1970; Converse and Cooper, 1979; Olson and Zanna, 1979, 1982; Gerard and White, 1983; Frey et al., 1984; Steele et al., 1993; Heine and Lehman, 1997; Lyubomirsky and Ross, 1999; Lieberman et al., 2001; Harmon-Jones and Harmon-Jones, 2002; Kitayama et al., 2004; Hoshino-Browne et al., 2005; Harmon-Jones et al., 2008; Imada and Kitayama, 2010; Lee and Schwarz, 2010). In these studies, results on relative difference in preference change between experimental conditions might be valid as the level of noise (and thus preference change explained by the artifact) should be no different across conditions as long as participants were randomly assigned into each experimental condition. However, without a proper control condition, any argument about absolute preference change would not be warranted (i.e., it is unclear whether or not significant dissonance reduction occurred in each condition). Furthermore, as discussed in the original critique (Chen and Risen, 2010), it remains possible that the moderating factors of interest may have affected the magnitude of noise in the rating and/or choice phases rather than a psychological process. This, in turn, could have produced the difference in the magnitude of spreading of alternatives across conditions (see Figure 4).

The same argument applies to past studies comparing preference change between two groups of individuals with different personalities (e.g., Gordon and Glass, 1970; Olson and Zanna, 1979, 1982; Steele et al., 1993; Lyubomirsky and Ross, 1999) or different cultural backgrounds (e.g., Heine and Lehman, 1997; Kitayama et al., 2004; Hoshino-Browne et al., 2005; Imada and Kitayama, 2010). Thus, although we believe that there is a fair chance that past findings of effects of different moderating variables hold, we cannot be sure without data, and the replication of these studies with a proper paradigm (e.g., blind choice or Rate-Rate-Choose paradigm) would be necessary.

### Neuroimaging studies using the paradigm

Three fMRI studies previously investigated which brain regions during the choice task (Jarcho et al., 2011; Kitayama et al., 2013) or the second rating task (Qin et al., 2011) tracks the degree of preference change on an item-by-item basis. Our simulation study showed that noise in the rating and choice phases alone could produce ostensible preference change. Thus, item-by-item preference change in the typical free-choice paradigm is at best very crude as a measure of true choice-induced preference change (or measure of choice justification). It is therefore unclear whether results reported in these studies (Jarcho et al., 2011; Qin et al., 2011; Kitayama et al., 2013) hold after addressing the criticism. Furthermore, because most neuroimaging studies (Sharot et al., 2009, 2010a; Jarcho et al., 2011; Qin et al., 2011; Kitayama et al., 2013) used the conventional free-choice paradigm, it remains unclear whether significant choice-induced preference change (or choice justification) occurred during each of these experiments. This renders it difficult to argue that brain activations reported in these studies are truly related to choice-induced preference change.

### Studies investigating individual differences in the spreading of alternatives

Past studies have also examined across-participant correlation between choice-induced preference change and other variables (e.g., Gawronski et al., 2007; Harmon-Jones et al., 2011; Qin et al., 2011). However, it is also likely that individual-level preference change does not correctly reflect how much an individual changed their true preference following choice (or how much an individual reduced cognitive dissonance), because individual differences in the magnitude of noise in each phase alone should have non-negligible influence on individual differences in reported preference change (see Figure 4). Data from Izuma et al. (2010) suggests that individual differences in preference changes, which are measured in the typical free-choice paradigm explain only about 28% of the total variance in true preference changes.^6^

### Summary

As reviewed above, the extent to which the criticism by Chen and Risen (2010) affects the validity of past studies varies depending on the experiment. Some findings could be explained without considering any change in true preference. In studies testing the effect of moderating factors, relative differences in preference change between experimental conditions may be valid, only if one can be certain that the moderating factors of interest did not affect the noise level in the rating and/or choice phases. Although some neuroimaging studies used item-by-item preference changes, it is doubtful that these changes reliably reflect change in true preference. Similarly, individual differences in preference change seem to be noisy as a measure of change in true preference. Again, this is not to say that all of these findings are false – our point is that it is difficult to evaluate the validity of each of these findings without appropriately controlling for the artifact pointed out by Chen and Risen (2010).

Recently, Kenworthy et al. (2011) conducted a meta-analysis to investigate whether results from different paradigms in cognitive dissonance research reflect a common mechanism. Interestingly, they found that a single variable (guilt) could explain effect sizes of all paradigms (such as induced compliance, insufficient justification, selective exposure, and disconfirmed expectancies) except for the free-choice paradigm. This result makes sense because effect sizes of past studies using the free-choice paradigm are confounded with the effect explained by the artifact, suggesting that at least some findings in studies using the free-choice paradigm are unreliable.

Finally, it should be noted that although a number of past studies did not address the criticism by Chen and Risen (2010) as stated above, not all findings reported in each of these studies are meaningless. Some of the studies included experiments using other paradigms in cognitive dissonance research (e.g., induced compliance) to which Chen and Risen’s (2010) criticism does not apply. Furthermore, even when the free-choice paradigm was used, some studies included findings that cannot be undermined by the methodological artifact. For example, Lyubomirsky and Ross (1999) investigated whether high school seniors’ evaluations of colleges they had applied to change after they themselves chose or rejected colleges and also after colleges rejected them. Although changes in the evaluations of colleges they themselves selected or rejected is susceptible to the artifact, the artifact does not affect changes in the evaluation of colleges that rejected them, because the choice was made by the college. They found that the evaluation of colleges that rejected them decreased, especially for happy individuals compared to unhappy individuals (Lyubomirsky and Ross, 1999). While detailed reviews and evaluations of all past studies that used the free-choice paradigm are well beyond the scope of the present paper, each reader should carefully examine how the criticism by Chen and Risen (2010) affects the overall conclusion of each of these previous studies that used the conventional free-choice paradigm.

## Conclusion

Chen and Risen (2010) pointed out an important methodological artifact in the more than 50-year-old free-choice paradigm. Our simulated study demonstrates the validity of their criticism and further shows how random noise in each phase of the free-choice paradigm differentially affects the artificial spreading of alternatives. In addition, three studies (Chen and Risen, 2010; Izuma et al., 2010; Sharot et al., 2012) empirically demonstrated that the artifact alone is sufficient to produce significant preference change. Taken together, these results suggest that the use of the traditional free-choice paradigm should be avoided in future research. Although some new studies that address the methodological flaw have already reported that choice does affect preference, these data do not validate all past findings. The criticism by Chen and Risen (2010) could still potentially undermine all past research in which the free-choice paradigm was used. Therefore, although we believe that at least some of results reported in previous studies hold even after addressing the problem, we agree with previous discussions (Chen and Risen, 2009; Sagarin and Skowronski, 2009a,b) and think that it is an empirical question. Thus, it is important to re-establish the effects of several moderating factors on the previously demonstrated process of choice-induced preference change. As the phenomenon of choice-induced preference change has been of great interest for psychologists, economists, and neuroscientists, questions addressed previously should be investigated in future research with an appropriate paradigm. Furthermore, as the effect size of choice-induced preference change is likely to be smaller than what was previously believed (see results of our meta-analysis), avoiding the “file drawer problem” would be important for correctly understanding this phenomenon (see Spellman, 2012).

## Conflict of Interest Statement

The authors declare that the research was conducted in the absence of any commercial or financial relationships that could be construed as a potential conflict of interest.

## Figures and Tables

**Figure A1:**
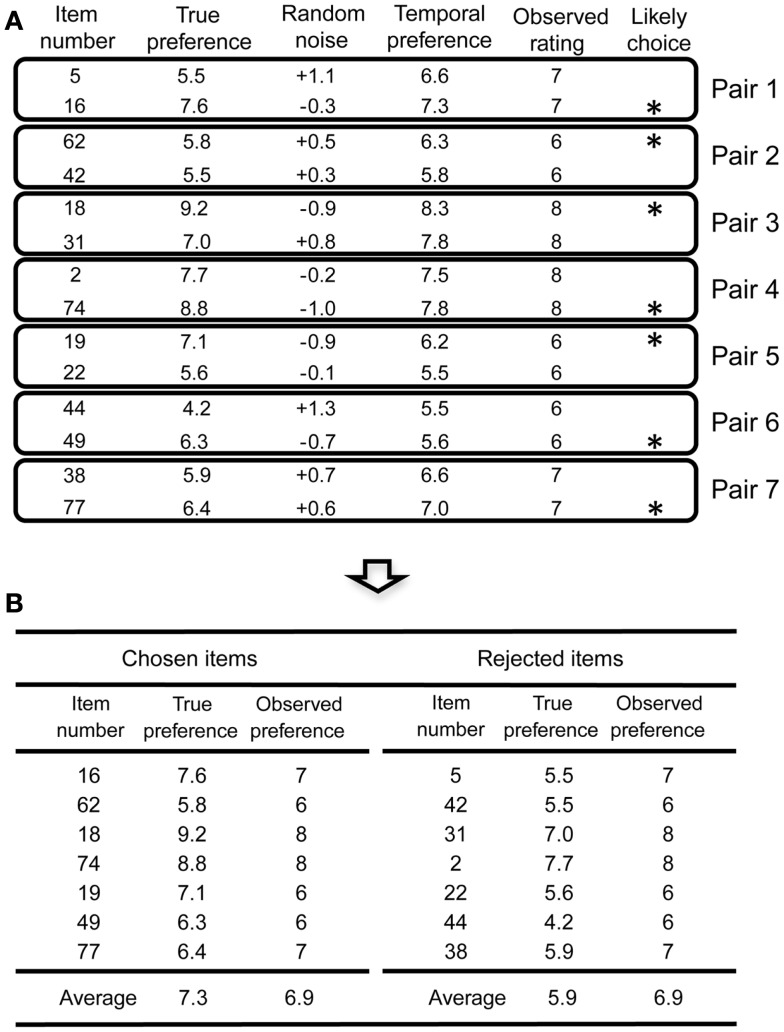
**(A)** Table including information about true preference, random noise, temporal preference, and observed ratings. Items are paired on the basis of observed ratings, but true preference is sometimes substantially different between the two items in a pair. Items with the asterisk are more likely to be chosen (i.e., they have higher true preference than their counterparts). **(B)** We sorted the items based on the likely choice suggested in **(A)**. Although the observed preference is equivalent, the chosen items have higher true preference than the rejected items.

## Footnotes

^1^There is another study reporting evidence for choice-induced preference change, which is yet to be published in a peer-reviewed journal (see Johansson et al., 2012). This study dissociated preference from choice by using a choice blindness paradigm (Johansson et al., 2005). In this paradigm, by using a magic trick, participants were led to believe that they chose an item that they had preferred less than the alternative (Johansson et al., 2012; see Henkel and Mather, 2007 for a conceptually similar manipulation and how beliefs about a past choice leads to memory modulation). This paradigm is similar to the blind choice paradigm used in Egan et al. (2010) and Sharot et al. (2010b) in the sense that participants’ choice no longer reflects their preference (in fact, this paradigm could be more conservative because the information revealed by choice biases preference change in the opposite direction. The item a participant thought he had selected is the one they actually preferred less, and thus the rating for the item is more likely to decrease rather than increase). One potentially important difference is that Johansson et al.’s choice blindness paradigm is likely to induce more cognitive dissonance because unlike the blind choice paradigm, participants think that their choice completely depended on their preference. Although this study is yet to be published, and its details are not yet available, it seems promising and the effect seems to be large (Johansson et al., 2012).

^2^The Rate-Rate-Choose condition was used as a control condition previously (Shultz et al., 1999). However, unlike our simulation study and three studies with the same control conditions (Chen and Risen, 2010; Izuma et al., 2010; Sharot et al., 2012), they did not find any spreading of alternatives in this control condition (Shultz et al., 1999).

^3^We ran a simulation study to test it and confirmed that their method of implicit choice is also susceptible to the artifact (data not shown).

^4^Alós-Ferrer et al. (2012) was not included in the meta-analysis because the exact effect size cannot be known from their robustness check analysis.

^5^Coppin et al. (2012) conducted three experiments to test the role of explicit memory for previous choices in preference change. In two of their experiments, they used a typical free-choice paradigm (i.e., the effect of information revealed by choices was not controlled), and in the final experiment, they used a version of the paradigm called “effort justification” to which Chen and Risen’s criticism does not apply. They found significant results in each of the three experiments and argued that because they found a similar effect in the paradigm where Chen and Risen’s criticism does not apply (third experiment), the results obtained in the first and second experiment are more than a mere artifact of the free-choice paradigm (Coppin et al., 2012). However, this is not true. The third experiment is completely independent from the first two experiments, and their results in the first and second experiments are simply inconclusive (the results are possible even without any change in true preference), as the experiments did not address the methodological problem.

^6^Preference changes measured in the conventional free-choice paradigm (PCmeasured) can be considered to include changes explained by the true effect of making a choice (PCtrue) and changes explained by the artifact (PCartifact; i.e., PCmeasured = PCtrue + PCartifact). To see how strong preference change by the artifact and true preference change by choice are related across individuals (association between PCmeasured and PCtrue), we re-analyzed the behavioral data from Izuma et al. (2010). Here, choice-induced preference change (PCtrue) is computed by taking the difference between preference changes seen in the conventional free-choice paradigm (Rate-Choose-Rate or Self-difficult condition) vs. preference changes produced purely by the artifact (the Rate-Rate-Choose or Post-experimental choice condition; i.e., PCtrue = PCmeasured − PCartifact). The correlation between PCmeasured and PCtrue was *r*(18) = 0.53 (*p* = 0.016). Although the correlation was statistically significant, the correlation coefficient of 0.53 indicates that preference change measured in the typical free-choice paradigm (PCmeasured) includes a considerable amount of noise (about 72% of the total variance) as a measure of individual difference in choice-induced preference change (PCtrue).

## Appendix

### Simulation details

In the simulation, hypothetical participants (*N* = 10) rated a set of 80 items on a 10-point scale (first rating; 1 = don’t like it at all; 10 = like it very much), then chose items among pairs (choice), and finally rated the items again on the same 10-point scale (second rating). For each participant, each item was assigned a “true preference score” on a continuous latent scale, which was randomly drawn from a normal distribution with mean 5.5 and standard deviation 2 (the mean and standard deviation were determined so that the true preference scores are reasonably distributed between 1 and 10). We assumed that true preference remains stable across the three phases. In the first rating phase, we added random noise to the true preference score to generate a “temporal preference” score and rounded that value to the nearest whole number. For example, if the true preference score was 5.5 and random noise was +1.1, then the observed rating was 7, rounded from a temporal preference score of 6.6. After the first rating, item pairs were presented that had been rated as equally attractive. That is, we paired items that received identical observed preference ratings in the 6–8 range. Figure A1 illustrates the item pairs created in one case of the simulation along with their true preference, random noise, and actual ratings. Note that, although each item in a pair received identical attractiveness ratings, their true preference is different. In addition, because random noise is added to the true preference score, some item pairs show substantial within-pair differences in the true preference scores.

In the choice task, random noise is again added to the true preference score, and we simply assumed that participants chose the item that has the higher temporal preference in this task. This procedure assures that participants are on average more likely to choose the item with the higher true preference score (the second assumption by Chen and Risen, 2010). As a result, chosen items are more likely to have higher true preference scores than rejected items. This point is illustrated in Figure A1B. We reordered the chosen and rejected items taken from hypothetical pairs depicted in Figure A1A (for illustrative purposes, we had participants simply choose the item with the higher true preference score in this figure). As is clear from the figure, although the observed ratings are identical between the chosen and rejected items, there is a bias in the true preference score that favors the chosen items.

In the second rating task, independent random noise was added again to the true preference score, and the same procedure with the first rating task was applied to obtain the observed ratings. Because chosen items have the higher true preference score, the chosen items are more likely to receive higher attractiveness ratings than the rejected items when items are rated again.

The simulation results are presented in Figure 3. In this simulation, noise in both the rating and choice phase was set at 0.5 (relative magnitude in comparison to the standard deviation of the true preference.

### Effects of scale coarseness

Scale coarseness influences the number of item pairs created for each participant (i.e., more fine-grained scale would create less item pairs that receive the same attractiveness ratings). However, it has little impact on the magnitude of the artifact, as the difference in true preference score in an item pair is irrelevant to whether the scale is coarse or not. To test our argument, we compared simulation results that used a 10-point scale and a 50-point scale (with 200 items; 1,000 replications). In the 10-point scale, items that ranged from 6 to 8 are paired and in the 50-point scale, items that ranged from 30 to 32 are paired. The other parameters are identical with those used in Figure 3. When a 10-point scale was used, the average spread of activation was 0.85. On the other hand, when a 50-point scale was used, the average spread of activation was 4.17. These two values are almost the same when the latter is transformed onto a 10-point scale (i.e., when the latter value was divided by 5). These results indicate that scale coarseness has little impact on the artifact.

## References

[B1] Alós-Ferrer C. Granić D. G. Shi F. Wagner A. K. (2012). Choices and preferences: evidence from implicit choices and response times. J. Exp. Soc. Psychol. 48, 1336–1342 10.1016/j.jesp.2012.07.004

[B2] Ariely D. Norton M. I. (2008). How actions create-not just reveal-preferences. Trends Cogn. Sci. (Regul. Ed.) 12, 13–16 10.1016/j.tics.2007.10.008 18063405

[B3] Bem D. J. (1967). Self-perception: an alternative interpretation of cognitive dissonance phenomena. Psychol. Rev. 74, 183–200 10.1037/h0025145 5342882

[B4] Brehm J. W. (1956). Post-decision changes in the desirability of choice alternatives. J. Abnorm. Soc. Psychol. 52, 384–389 10.1037/h0041006 13318848

[B5] Brehm J. W. Cohen A. R. (1959). Re-evaluation of choice alternatives as a function of their number and qualitative similarity. J. Abnorm. Soc. Psychol. 58, 373–378 10.1037/h0040493 13653888

[B6] Brehm J. W. Jones R. A. (1970). Effect on dissonance of surprise consequences. J. Exp. Soc. Psychol. 6, 420–431 10.1016/0022-1031(70)90053-3

[B7] Brehm J. W. Wicklund R. A. (1970). Regret and dissonance reduction as a function of postdecision salience of dissonant information. J. Pers. Soc. Psychol. 14, 1–7 10.1037/h0028616 5435535

[B8] Brock T. C. (1963). Effects of prior dishonesty on postdecision dissonance. J. Abnorm. Psychol. 66, 325–331 10.1037/h0047653 14015625

[B9] Chen M. K. Risen J. L. (2009). Is choice a reliable predictor of choice? A comment on Sagarin and Skowronski. J. Exp. Soc. Psychol. 45, 425–427 10.1016/j.jesp.2008.07.024

[B10] Chen M. K. Risen J. L. (2010). How choice affects and reflects preferences: revisiting the free-choice paradigm. J. Pers. Soc. Psychol. 99, 573–594 10.1037/a0020217 20658837

[B11] Cohen J. (1988). Statistical Power Analysis for the Behavioral Sciences. Hillsdale, NJ: Lawrence Erlbaum

[B12] Converse J. Cooper J. (1979). Importance of decisions and free-choice attitude-change: a curvilinear finding. J. Exp. Soc. Psychol. 15, 48–61 10.1016/0022-1031(79)90017-9

[B13] Coppin G. Delplanque S. Cayeux I. Porcherot C. Sander D. (2010). I’m no longer torn after choice: how explicit choices implicitly shape preferences of odors. Psychol. Sci. 21, 489–493 10.1177/0956797610364115 20424088

[B14] Coppin G. Delplanque S. Porcherot C. Cayeux I. Sander D. (2012). When flexibility is stable: implicit long-term shaping of olfactory preferences. PLoS ONE 7:e37857 10.1371/journal.pone.0037857 22761661 PMC3380896

[B15] Deutsch M. Krauss R. M. Rosenau N. (1962). Dissonance of defensiveness. J. Pers. 30, 16–28 13885858

[B16] Dunlap W. P. Cortina J. M. Vaslow J. B. Burke M. J. (1996). Meta-analysis of experiments with matched groups or repeated measures designs. Psychol. Methods 1, 170–177 10.1037/1082-989X.1.2.170

[B17] Egan L. C. Bloom P. Santos L. R. (2010). Choice-induced preferences in the absence of choice: evidence from a blind two choice paradigm with young children and capuchin monkeys. J. Exp. Soc. Psychol. 46, 204–207 10.1016/j.jesp.2009.08.014

[B18] Egan L. C. Santos L. R. Bloom P. (2007). The origins of cognitive dissonance – evidence from children and monkeys. Psychol. Sci. 18, 978–983 10.1111/j.1467-9280.2007.02012.x 17958712

[B19] Festinger L. (1957). A Theory of Cognitive Dissonance. Stanford: Stanford University Press

[B20] Frey D. Kumpf M. Irle M. Gniech G. (1984). Re-evaluation of decision alternatives dependent upon the reversibility of a decision and the passage of time. Eur. J. Soc. Psychol. 14, 447–450 10.1002/ejsp.2420140410

[B21] Gawronski B. Bodenhausen G. V. Becker A. P. (2007). I like it, because I like myself: associative self-anchoring and post-decisional change of implicit evaluations. J. Exp. Soc. Psychol. 43, 221–232 10.1016/j.jesp.2006.04.001

[B22] Gerard H. B. White G. L. (1983). Post-decisional reevaluation of choice alternatives. Pers. Soc. Psychol. Bull. 9, 365–369 10.1177/0146167283093006

[B23] Gordon A. Glass D. C. (1970). Choice ambiguity, dissonance, and defensiveness. J. Pers. 38, 264–272 10.1111/j.1467-6494.1970.tb00008.x 5427614

[B24] Greenwald H. J. (1969). Dissonance and relative versus absolute attractiveness of decision alternatives. J. Pers. Soc. Psychol. 11, 328–333 10.1037/h0027369 5787021

[B25] Harmon-Jones C. Schmeichel B. J. Inzlicht M. Harmon-Jones E. (2011). Trait approach motivation relates to dissonance reduction. Soc. Psychol. Personal. Sci. 2, 21–28 10.1177/1948550610379425

[B26] Harmon-Jones E. Harmon-Jones C. (2002). Testing the action-based model of cognitive dissonance: the effect of action orientation on postdecisional attitudes. Pers. Soc. Psychol. Bull. 28, 711–723 10.1177/0146167202289001

[B27] Harmon-Jones E. Harmon-Jones C. Fearn M. Sigelman J. D. Johnson P. (2008). Left frontal cortical activation and spreading of alternatives: tests of the action-based model of dissonance. J. Pers. Soc. Psychol. 94, 1–15 10.1037/0022-3514.94.1.1 18179314

[B28] Heine S. J. Lehman D. R. (1997). Culture, dissonance, and self-affirmation. Pers. Soc. Psychol. Bull. 23, 389–400 10.1177/0146167297234005

[B29] Henkel L. A. Mather M. (2007). Memory attibutions for choices: how beliefs shape our memories. J. Mem. Lang. 57, 163–176 10.1016/j.jml.2006.08.012

[B30] Holden S. (2013). Do choices affect preferences? some doubts and new evidence. J. App. Soc. Psychol. 43, 83–94 10.1111/j.1559-1816.2012.00983.x

[B31] Hoshino-Browne E. Zanna A. S. Spencer S. J. Zanna M. P. Kitayama S. Lackenbauer S. (2005). On the cultural guises of cognitive dissonance: the case of easterners and westerners. J. Pers. Soc. Psychol. 89, 294–310 10.1037/0022-3514.89.3.294 16248715

[B32] Imada T. Kitayama S. (2010). Social eyes and choice justification: culture and dissonance revisited. Soc. Cogn. 28, 589–608 10.1521/soco.2010.28.5.589

[B33] Izuma K. Matsumoto M. Murayama K. Samejima K. Sadato N. Matsumoto K. (2010). Neural correlates of cognitive dissonance and choice-induced preference change. Proc. Natl. Acad. Sci. U.S.A. 107, 22014–22019 10.1073/pnas.1011879108 21135218 PMC3009797

[B34] Jarcho J. M. Berkman E. T. Lieberman M. D. (2011). The neural basis of rationalization: cognitive dissonance reduction during decision-making. Soc. Cogn. Affect. Neurosci. 6, 460–467 10.1093/scan/nsq054 20621961 PMC3150852

[B35] Johansson P. Hall L. Charter N. (2012). “Preference change through choice,” in Neuroscience of Preference and Choice: Cognitive and Neural Mechanisms, eds. Dolan R. J. Sharot T. (London: Academic Press), 121–142

[B36] Johansson P. Hall L. Sikstrom S. Olsson A. (2005). Failure to detect mismatches between intention and outcome in a simple decision task. Science 310, 116–119 10.1126/science.1111709 16210542

[B37] Kenworthy J. B. Miller N. Collins B. E. Read S. J. Earleywine M. (2011). A trans-paradigm theoretical synthesis of cognitive dissonance theory: illuminating the nature of discomfort. Eur. Rev. Soc. Psychol. 22, 36–113 10.1080/10463283.2011.580155

[B38] Kimel S. Y. Grossmann I. Kitayama S. (2012). When gift-giving produces dissonance: effects of subliminal affiliation priming on choices for one’s self versus close others. J. Exp. Soc. Psychol. 48, 1221–1224 10.1016/j.jesp.2012.05.012

[B39] Kitayama S. Chua H. F. Tompson S. Han S. (2013). Neural mechanisms of dissonance: an fMRI investigation of choice justification. Neuroimage 69, 206–212 10.1016/j.neuroimage.2012.11.034 23238432

[B40] Kitayama S. Snibbe A. C. Markus H. R. Suzuki T. (2004). Is there any “free” choice? Self and dissonance in two cultures. Psychol. Sci. 15, 527–533 10.1111/j.0956-7976.2004.00714.x 15270997

[B41] Lawler E. E. Kuleck W. J. Rhode J. G. Sorensen J. E. (1975). Job choice and post decision dissonance. Organ. Behav. Hum. Perform. 13, 133–145 10.1016/0030-5073(75)90009-4

[B42] Lee S. W. Schwarz N. (2010). Washing away postdecisional dissonance. Science 328, 709 10.1126/science.1187722 20448177

[B43] Leotti L. A. Iyengar S. S. Ochsner K. N. (2010). Born to choose: the origins and value of the need for control. Trends Cogn. Sci. (Regul. Ed.) 14, 457–463 10.1016/j.tics.2010.08.001 20817592 PMC2944661

[B44] Lieberman M. D. Ochsner K. N. Gilbert D. T. Schacter D. L. (2001). Do amnesics exhibit cognitive dissonance reduction? The role of explicit memory and attention in attitude change. Psychol. Sci. 12, 135–140 10.1111/1467-9280.00323 11340922

[B45] Lyubomirsky S. Ross L. (1999). Changes in attractiveness of elected, rejected, and precluded alternatives: a comparison of happy and unhappy individuals. J. Pers. Soc. Psychol. 76, 988–1007 10.1037/0022-3514.76.6.988 10402682

[B46] Olson J. M. Zanna M. P. (1979). New look at selective exposure. J. Exp. Soc. Psychol. 15, 1–15 10.1016/0022-1031(79)90014-3

[B47] Olson J. M. Zanna M. P. (1982). Repression-sensitization differences in responses to a decision. J. Pers. 50, 46–57 10.1111/j.1467-6494.1982.tb00744.x

[B48] Qin J. Kimel S. Kitayama S. Wang X. Yang X. Han S. (2011). How choice modifies preference: neural correlates of choice justification. Neuroimage 55, 240–246 10.1016/j.neuroimage.2010.11.076 21130888

[B49] Risen J. Chen M. K. (2010). How to study choice-induced attitude change: strategies for fixing the free-choice paradigm. Soc. Personal. Psychol. Compass 4, 1151–1164 10.1111/j.1751-9004.2010.00323.x

[B50] Sagarin B. J. Skowronski J. J. (2009a). The implications of imperfect measurement for free-choice carry-over effects: reply to M. Keith Chen’s (2008) “Rationalization and cognitive dissonance: do choices affect or reflect preferences?” J. Exp. Soc. Psychol. 45, 421–423 10.1016/j.jesp.2008.08.024

[B51] Sagarin B. J. Skowronski J. J. (2009b). In pursuit of the proper null: reply to Chen and Risen (2009). J. Exp. Soc. Psychol. 45, 428–430 10.1016/j.jesp.2008.08.024

[B52] Sharot T. De Martino B. Dolan R. J. (2009). How choice reveals and shapes expected hedonic outcome. J. Neurosci. 29, 3760–3765 10.1523/JNEUROSCI.4972-08.2009 19321772 PMC2675705

[B53] Sharot T. Fleming S. M. Yu X. Koster R. Dolan R. J. (2012). Is choice-induced preference change long lasting? Psychol. Sci. 23, 1123–1129 10.1177/0956797612438733 22933456 PMC3802118

[B54] Sharot T. Shiner T. Dolan R. J. (2010a). Experience and choice shape expected aversive outcomes. J. Neurosci. 30, 9209–9215 20610755 10.1523/JNEUROSCI.4770-09.2010 PMC2923025

[B55] Sharot T. Velasquez C. M. Dolan R. J. (2010b). Do decisions shape preference? Evidence from blind choice. Psychol. Sci. 21, 1231–1235 10.1177/0956797610379235 20679522 PMC3196841

[B56] Shultz T. R. Leveille E. Lepper M. R. (1999). Free choice and cognitive dissonance revisited: choosing “lesser evilts” versus “greater goods.” Pers. Soc. Psychol. Bull. 25, 40–48 10.1177/0146167299025001004

[B57] Spellman B. A. (2012). Introduction to the special section: data, data, everywhere … especially in my file drawer. Perspect. Psychol. Sci. 7, 58–59 10.1177/1745691612465075 26168423

[B58] Steele C. M. Spencer S. J. Lynch M. (1993). Self-image resilience and dissonance: the role of affirmational resources. J. Pers. Soc. Psychol. 64, 885–896 10.1037/0022-3514.64.6.885 8326471

[B59] Vroom V. H. (1966). Organizational choice – study of predecision and postdecision processes. Organ. Behav. Hum. Perform. 1, 212–225 10.1016/0030-5073(66)90005-5

[B60] Walster E. Berschei E. Barclay A. M. (1967). A determinant of preference among modes of dissonance reduction. J. Pers. Soc. Psychol. 7, 211–216 10.1037/h0024992

[B61] Walster G. W. Walster E. (1970). Choice between negative alternatives – dissonance reduction or regret. Psychol. Rep. 26, 995–1005 10.2466/pr0.1970.26.3.995

[B62] West S. Jett S. E. Beckman T. Vonk J. (2010). The phylogenetic roots of cognitive dissonance. J. Comp. Psychol. 124, 425–432 10.1037/a0019932 20836593

